# Secondary-Ion Mass Spectrometry Analysis of Pentosan Polysulfate

**DOI:** 10.1021/acsomega.6c05275

**Published:** 2026-09-10

**Authors:** Andrew L. Hook, Anna M. Kotowska, Huw E. L. Williams, Holger Wondraczek

**Affiliations:** † Advanced Materials and Healthcare Technology, University of Nottingham, NG7 2RD Nottingham, U.K.; ‡ Biodiscovery Institute, Faculty of Science, University of Nottingham, NG7 2RD Nottingham, U.K.; § HV-Polysaccharides GmbH & Co. KG, Am Amselberg 1, 07751 Bucha, Germany; ∥ Fraunhofer Center for Chemical-Biotechnological Processes CBP, Am Haupttor (Gate 12, Building 1251), 06237 Leuna, Germany

## Abstract

Pentosan polysulfate sodium (PPS) is a glycosaminoglycan mimic that is the only current active pharmaceutical ingredient (API) for the oral treatment of interstitial cystitis. PPS is an inherently complex API due to variations in chain length, backbone structure, sulfation pattern, and end group, making discriminating between batch-to-batch as well as manufacturer-to-manufacturer differences difficult. In this study, high-throughput liquid handling and time-of-flight secondary-ion mass spectrometry (ToF-SIMS) are used to enable the rapid chemical analysis (450 samples in 6 h) of small amounts (<150 ng) of PPS. Chemical discrimination between PPS produced by different manufacturers and, in one case, batch-to-batch variation of the same manufacturer were achieved. Analysis of samples using an OrbiSIMS instrument is used to provide confident, putative assignment of the discriminating ions to enable structural interpretation of the variance observed between samples. Together with analysis of samples spiked with NaSO_4_, free sulfate, degree of sulfation along with the sulfate pattern are implicated as key differences between manufacturers/batches. This approach is likely to be highly beneficial for the rapid quality control of PPS.

## Graphical abstract

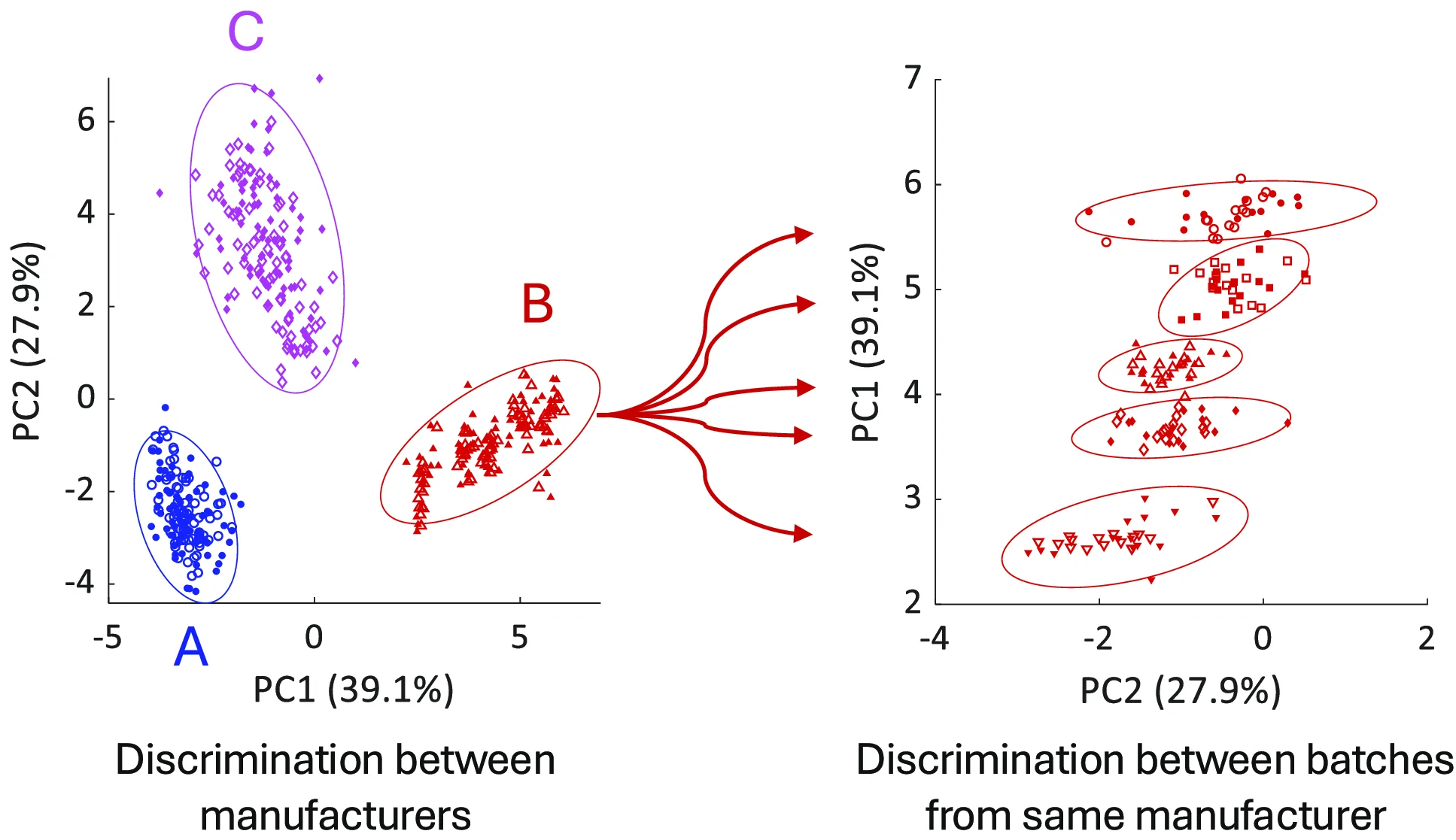

## 1 Introduction

Pentosan polysulfate sodium (PPS) is considered a functional glycosaminoglycan mimetic possessing a variety of biological properties, making it an interesting compound for different pharmaceutical applications. For example, it is currently the only active pharmaceutical ingredient (API) approved by US-FDA for the treatment of interstitial cystitis, an orphan disease characterized by painful urinary symptoms.^1,2^ Prolonged use of PPS has recently been associated with retinal toxicity leading to maculopathy.^3^ PPS is also a promising drug candidate in the treatment of chronic pelvic pain syndrome,^4^ mucopolysaccharidoses type VI,^5^ diabetic nephropathy,^6,7^ and iron imbalance due to hepcidin dysregulation^8^ as well as for prevention of tumor growth^9^ and alphavirus-induced arthritis.^10^ It was also explored as a potential treatment in the context of SARS-CoV-2 infections.^11–13^

Commercially, PPS is obtained by an exhaustive, nonselective O-sulfation of hardwood derived glucuronoxylans.^14^ As a result of the natural origin of the glucuronoxylans as well as due to their chemical modification, PPS possesses several aspects of molecular heterogenicity making it a complex API:

1. It is a mixture of molecules with a varying chain length of the β-1-4-linked xylose backbone.
2. The content of 4-O-methyl glucuronic acid residues intermittently attached to the backbone via an α-1→2 linkage can vary.^15^
3. Native glucuronoxylans contain O-acetyl substituents, which can be carried forward into PPS.^16^
4. The degree of sulfation may vary depending on the reaction conditions.^17^
5. The specific pathway of O-sulfation can cause certain modifications of the end groups of the polysaccharide chain.^18,19^

An in-depth analysis of these structural aspects can be achieved using nuclear magnetic resonance (NMR) spectroscopy and liquid chromatography coupled with mass spectrometry (LC-MS).^20–24^ These methods provide a detailed picture of the structural heterogeneity of PPSs (Figure 1). However, this approach is typically slow, requires substantial amounts of sample, and lacks sensitivity. In the context of the ongoing exploration of PPS in various clinical applications, additional analytical methods would be beneficial that could rapidly analyze small quantities of materials with high sensitivity detection of batch-to-batch variations.^25^ For this purpose, time-of-flight secondary-ion mass spectrometry (ToF-SIMS) is a promising method. It was recently shown that ToF-SIMS can discriminate between different glycosaminoglycans with high sensitivity, including pharmaceutical heparins derived either from bovine or porcine sources.^26,27^ In this study, <200 ng of material (no pre-processing required) was needed to detect heparins from different animal sources within porcine-derived heparin to a sensitivity as low as 0.001 wt %. By combining with liquid handling systems that allowed the creation of microarrays of materials, hundreds of samples were successfully analyzed over a few hours. High mass resolution spectra can be acquired using the Hybrid SIMS instrument equipped with an Orbitrap mass analyzer,^28^ which has been successfully used to characterize polysaccharides within *Pseudomonas aeruginosa* biofilm.^29^

**Figure 1:**
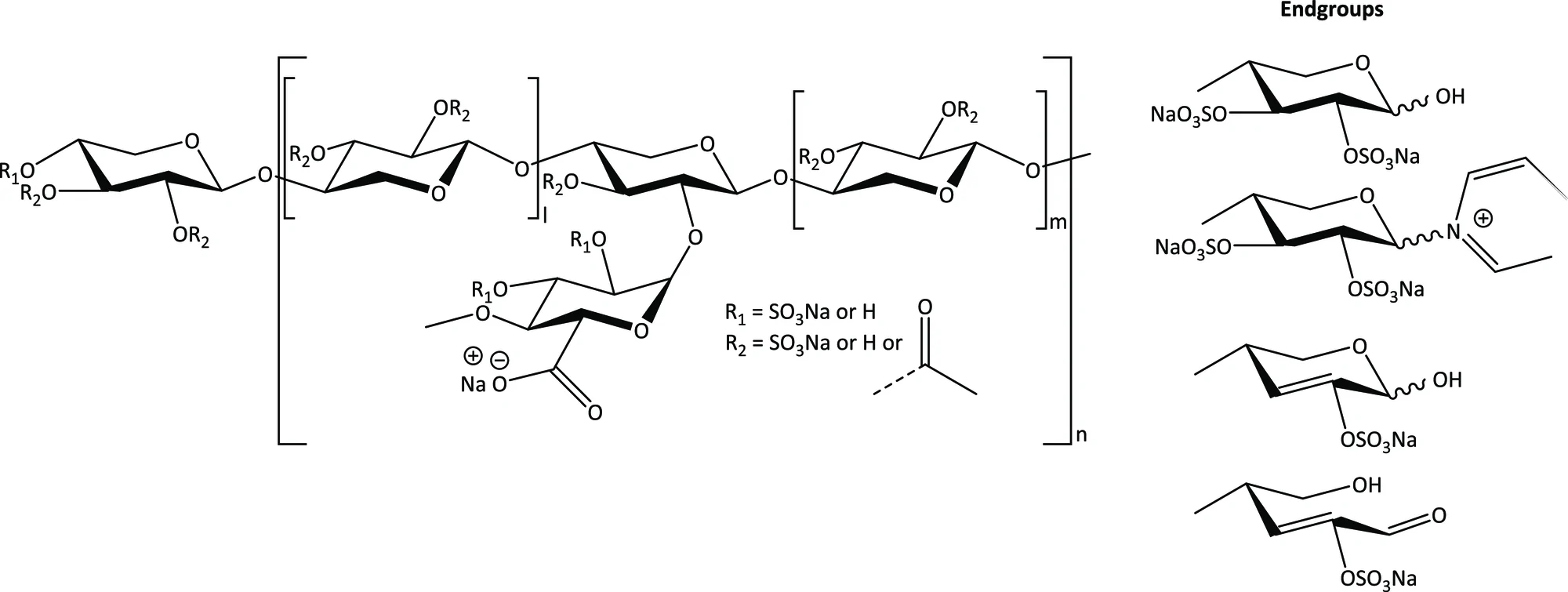
Chemical structure of PPS. Used with permission.^20^

In the present work, we investigated the utility of ToF-SIMS in the analysis of PPS, with an emphasis on being able to assess differences between samples. For that purpose, a microarray of several different batches of PPS obtained from drug capsules of three different manufacturers was analyzed. Subsequently, the discriminative power of ToF-SIMS with respect to batch-to-batch as well as manufacturer-to-manufacturer differences was established based on principal component analysis (PCA).

## 2 Experimental Section

### 2.1 Materials

PPS samples were obtained by aqueous extraction from the drug capsules of three different manufacturers. The individual capsules were taken from five different batches of the drug product from each manufacturer. As per the label claim, the drug products contained the same kind of water-insoluble excipient, namely, microcrystalline cellulose and magnesium stearate, in similar quantities. The identity of the excipients was confirmed by solid-state NMR (data not shown).

For the extraction of PPS, the content of one capsule was suspended in 10 mL of water and stirred at room temperature (20–25 °C) for 2 h. Subsequently, the water-insoluble fraction was removed by filtration using a 0.22 μm mixed cellulose ester membrane (Milipore GSWP02500). The filter cake and membrane were washed with another 5 mL of water. The pooled aqueous filtrate and washing solution were submitted to freeze drying yielding 100 ± 3% of the label claim of PPS.

Poly-l-lysine-coated slides (Poly-Prep, Sigma-Aldrich) were used as received.

### 2.2 NMR Analysis

PPS samples (45-55 mg) were dissolved in 0.6 mL of deuteriumoxide. NMR experiments were performed on a Bruker 800 MHz Avance Neo spectrometer using a 5 mm QCI Cryoprobe. The temperature was set at 25 °C (298.15 K). Proton spectra were acquired using 128 scans of 32K points with a spectral width of 13 ppm. The free induction decays (FID) were multiplied by an exponential weighting function with a line broadening of 2 Hz before Fourier transformation. After calibration there were small variations to the reported values,^24^ likely due to temperature and pH differences between experiments. To allow comparison, we calibrated our spectra based upon the Xyl_α_ (py), 1MGA_Xylα_, and ^1^Xyl_2MGA_ peaks within the anomeric region of the proton and carbon spectra. Additionally two-dimensional NMR spectra (^13^C-HSQC) were acquired to confirm proton identity. Spectra were acquired: 64 transients and with 2k points in the direct dimension and with between 256 and 1024 points in the indirect dimension. Data were subsequently apodised with sine squared window functions and zero-filled by at least a factor of two.

### 2.3 Preparation of Spiked Samples

To provide further structural insights into key ions identified via PCA, three PPS samples at 3.75 mg/mL, one from each manufacturer, were spiked with sodium sulfate (Scientific Laboratory Supplies, 92%, NaSO_4_) from 0.1 M stock solutions to create final concentrations of 0, 2.5, or 10 mM.

### 2.4 Microarray Preparation

Arrays were prepared using an s11 sciFLEXARRAYER dispensing system (Scienion) using a glass piezo dispense capillary (P-2020, Scienion), as previously described.^26^ Drop volumes were ≈300 pL. Print runs were conducted at a relative humidity of 65–70 % at room temperature (20–25 °C). PPS solutions (5 or 3.75 mg/mL for spiked samples) in water were dispensed from a polystyrene 384-well plate (Corstar). A total of 30 nL of each sample were dispensed, equating to 150 ng. Samples were allowed to dry at ambient conditions before analysis.

### 2.5 ToF-SIMS Analysis

Time-of-flight secondary-ion mass spectrometry measurements were conducted as previously described^26,27^ using a ToF-SIMS IV (IONTOF GmbH, Münster, Germany) instrument, a 25 keV Bi_3_^+^ primary ion source (pulsed target current of >0.3 pA). Sampling pixel density was 128 pixels per mm, with 15 shots per pixel and an ion dose of 2.45 × 10^11^ ions per cm^2^. Both positive and negative secondary-ion spectra were collected (mass resolution of >7000 at *m*/*z* = 29) with charge compensation using a low energy (20 eV) electron floodgun. Analysis conditions for both the ToF and OrbiTrap mass analyzers were optimized to ensure the detectors were not saturated and peak intensities were assessed to ensure maximum signals were below the saturation limit of the detectors used. Patch areas of 0.5 × 0.5 mm were acquired sequentially in order to capture data from the entire microarray area. A peak list previously derived from a range of glycosaminoglycan (GAG) samples that included both sulfated and non-sulfated ions was applied.^26^ Regions associated with each sample were extracted and recalibrated before quantification of the counts per peak.

### 2.6 OrbiSIMS Analysis

A single replicate from each PPS manufacturer was prepared for OrbiSIMS analysis by pipetting 2 μl of 5 mg/mL PPS solution onto a PLL-coated slide and allowing to air dry at ambient conditions. For the acquisition of the OrbiSIMS spectra, a 30 keV Bi_3_^+^ primary ion source was used. The Bi ion gun was operated in long pulse mode (pulse width was 10%), the primary ion beam current was 2.3 nA. The Q Exactive Orbitrap mass spectrometer depth profile was run on the area of 200 × 200 μm using random raster mode with crater size 280 × 280 μm. The cycle time was set to 400 μs. Optimal target potential varied for different samples and was approximately +68 V. Argon gas flooding was in operation in order to aid charge compensation. Pressure in the main chamber was maintained at 9.0 × 10^–7^ mbar. The spectra were collected in negative polarity, in mass range *m*/*z* 75–1000. The injection time was set to 500 ms. One area was analyzed on each sample and each measurement lasted 30 scans. Mass-resolving power was set to 240,000 at *m*/*z* 200.

### 2.7 Multivariate Analysis

PCA was conducted as previously described.^26^ Datasets were variance scaled and mean-centered prior to analysis. Replicate measurements were split into training (50%) and validation (50%) sets. A sparse dataset was created as previously^26^ described using sequential random feature addition (RFA), with a cost function of separation between samples, and then random feature elimination (RFE), using a cost function of overlap of confidence ellipses. After each feature elimination, the resulting models were checked to ensure the validation datasets fell within the 95% confidence limits of the training datasets. The minimum number of variables required to separate the samples was selected.

For comparison between the OrbiSIMS and ToF-SIMS data, the OrbiSIMS spectra of the three representative samples from each manufacturer were visually inspected in the *m*/*z* ranges associated with the ToF-SIMS peaks selected within the final sparse dataset for the PCA model. In all cases, a high-intensity peak that aligned with the *m*/*z* value for each ToF-SIMS peak and could be assigned to a chemical structure(s) potentially derived from PPS was identified in at least one of the PPS samples.

## 3 Results and Discussion

### 3.1 NMR Analysis

A single PPS sample from each manufacturer underwent high resolution 2D ^1^H–^13^C HSQC NMR analysis (Figure 2). Each NMR analysis required approximately 50 mg of sample and took approximately 5 h to analyze due to the complexity of the PPS samples. The 1D ^1^H NMR spectra for the three samples displayed similar spectral features to each other and to PPS samples analyzed previously,^20,22,24^ confirming the identity of the PPS samples we were analyzing. There were some differences noted between different manufacturers. The sample from manufacturer A had a reduced acetyl signal (2–2.3 ppm)^20^ (Figure 2A), whilst all samples showed small differences in pyridinium and aldehyde protons associated with end groups (8–9.4 ppm)^22^ (Figure 2C). There were also notable differences in the anomeric region (4.9–6.4 ppm), with peaks at ≈5.8 and 5.9 ppm only present in the sample from manufacturer A and the peak at ≈6.0 ppm absent from the sample from manufacturer B. The differences in this region of the spectra were explored in more detail using HSQC NMR (Figure 3).

**Figure 2:**
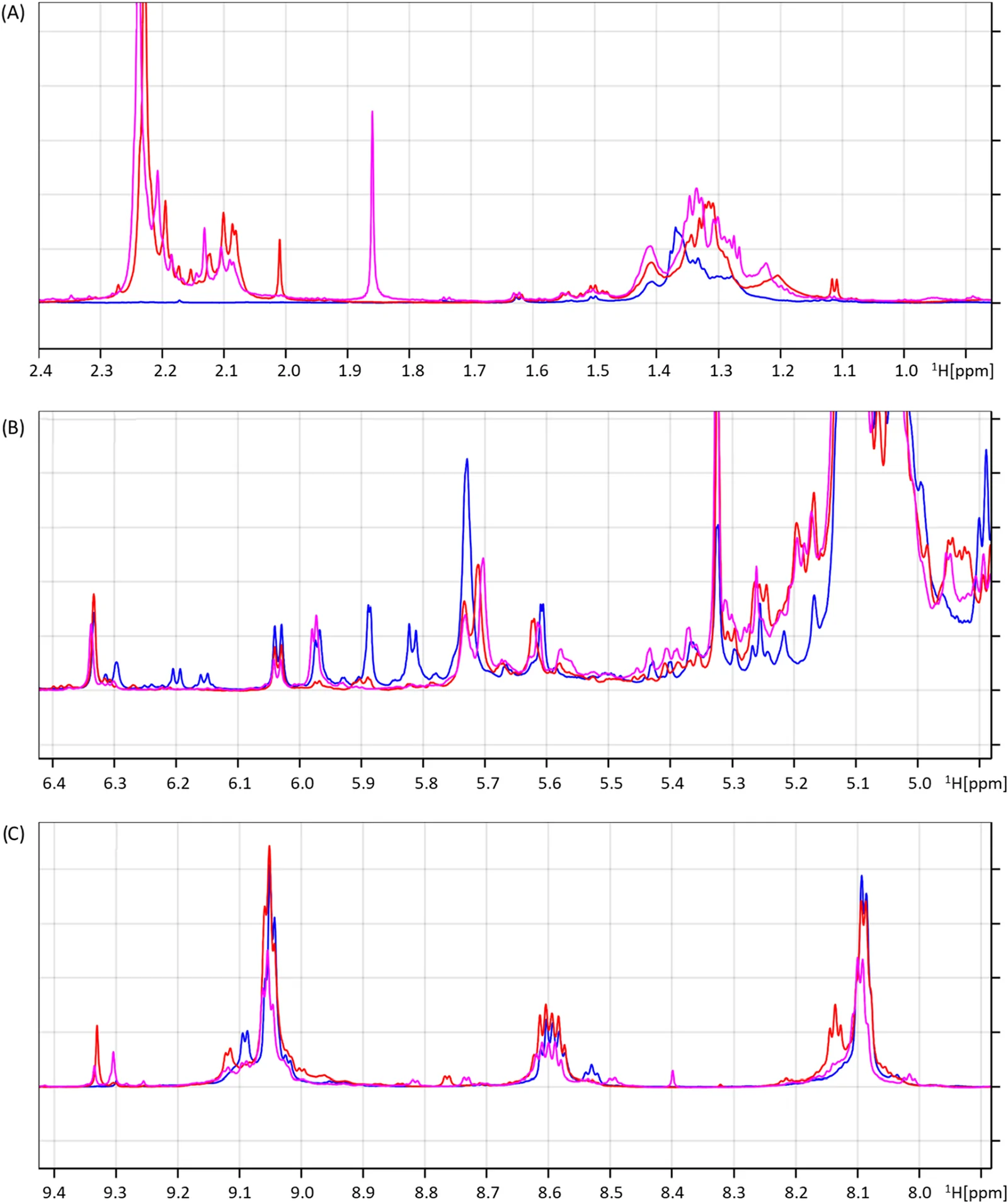
1H NMR spectra of PPS samples from manufacturers A (blue square), B (red square), and C (pink square), showing (A) acetyl (0.9–2.4 ppm), (B) anomeric (4.9–6.4 ppm), and (C) aldehyde or pyridinium (8–9.4 ppm) proton resonance regions.

**Figure 3:**
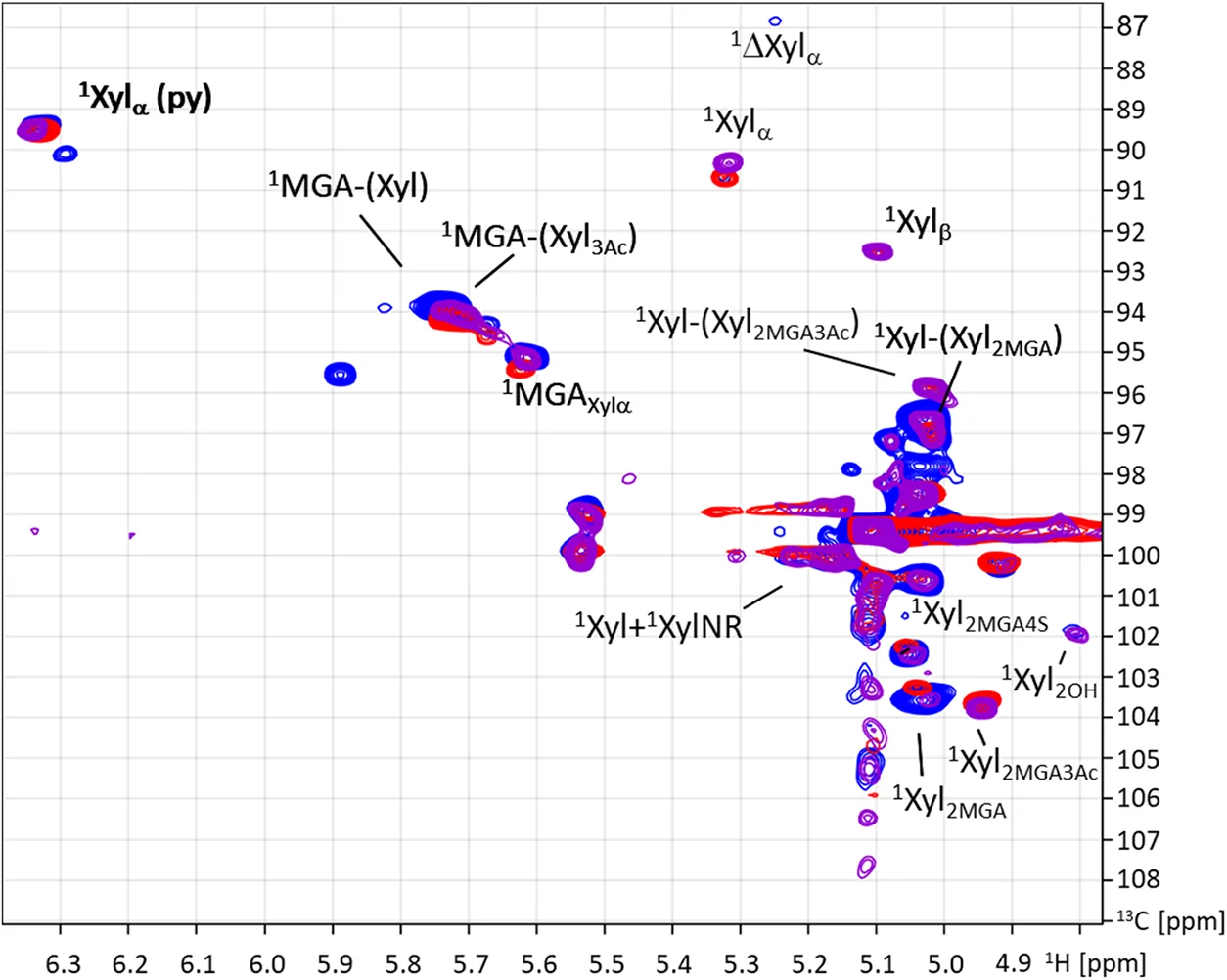
HSQC NMR region of PPS samples from manufacturers A (blue square), B (red square), and C (pink square). Xyl = xylose-2,3-di-O-sulfate (unless otherwise specified), MGA = 4-O-methyl-2,3-di-O-sulfated glucuronic acid, Ac = acetyl, Me = methyl, Py = pyridine, ΔXylred – C2–C3 double bond, ΔXylred­(CHO) – open ring aldehyde with C2–C3 double bond. Assignments from.^24^

The assignment of peaks within the HSQC NMR analysis of samples was based upon results previously reported.^24^ Although material from all of the 3 manufacturers showed substantial overlap with each other and with samples previously analyzed, notable qualitative differences were observed between the different PPS samples. Only material from manufacturer A exhibited peaks associated with 2,3-unsaturated xylose-2-O-sulfate (^1^ΔXyl_α_) but was missing the peaks associated with xylose-2,3-di-O-sulfate (Xyl) branched with acetylated 4-O-methyl- glucuronic acid (MGA) (^1^Xyl_2MGA3Ac_ and ^1^Xyl-(Xyl_2MGA3Ac_)). This was consistent with the reduced acetyl signal observed in the region of 2–2.3. Although samples B and C showed almost complete overlap, the signal for ^1^Xyl_2OH_ was absent from the sample from manufacturer B. This suggests higher levels of sulfation in this sample.

### 3.2 Comparison of ToF-SIMS and OrbiSIMS Analysis of PPS Samples

Microarrays are an important tool for rapid and miniaturized biomaterials discovery, drug formulation, and analysis of biomolecular interactions.^30–33^ To enable the high-throughput assessment of PPS samples, approximately 150 ng of the extracted water-soluble components from 5 different batches from 3 different PPS drug product manufacturers were printed using inkjet printing onto PLL-coated slides, with 30 technical replicates printed per sample. This array was analyzed by ToF-SIMS as a total area scan, and spectra associated with each sample were extracted using region of interest analysis. One biological replicate for each manufacturer was also pipetted and dried onto a slide to enable the OrbiSIMS analysis. Spectra from 450 separate samples were acquired over a period of approximately 6 h, equating to less than 1 min analysis time per sample. For reference, a single replicate from each manufacturer was analyzed using OrbiSIMS.^28^ Representative SIMS spectra are shown in Figure 4.

**Figure 4:**
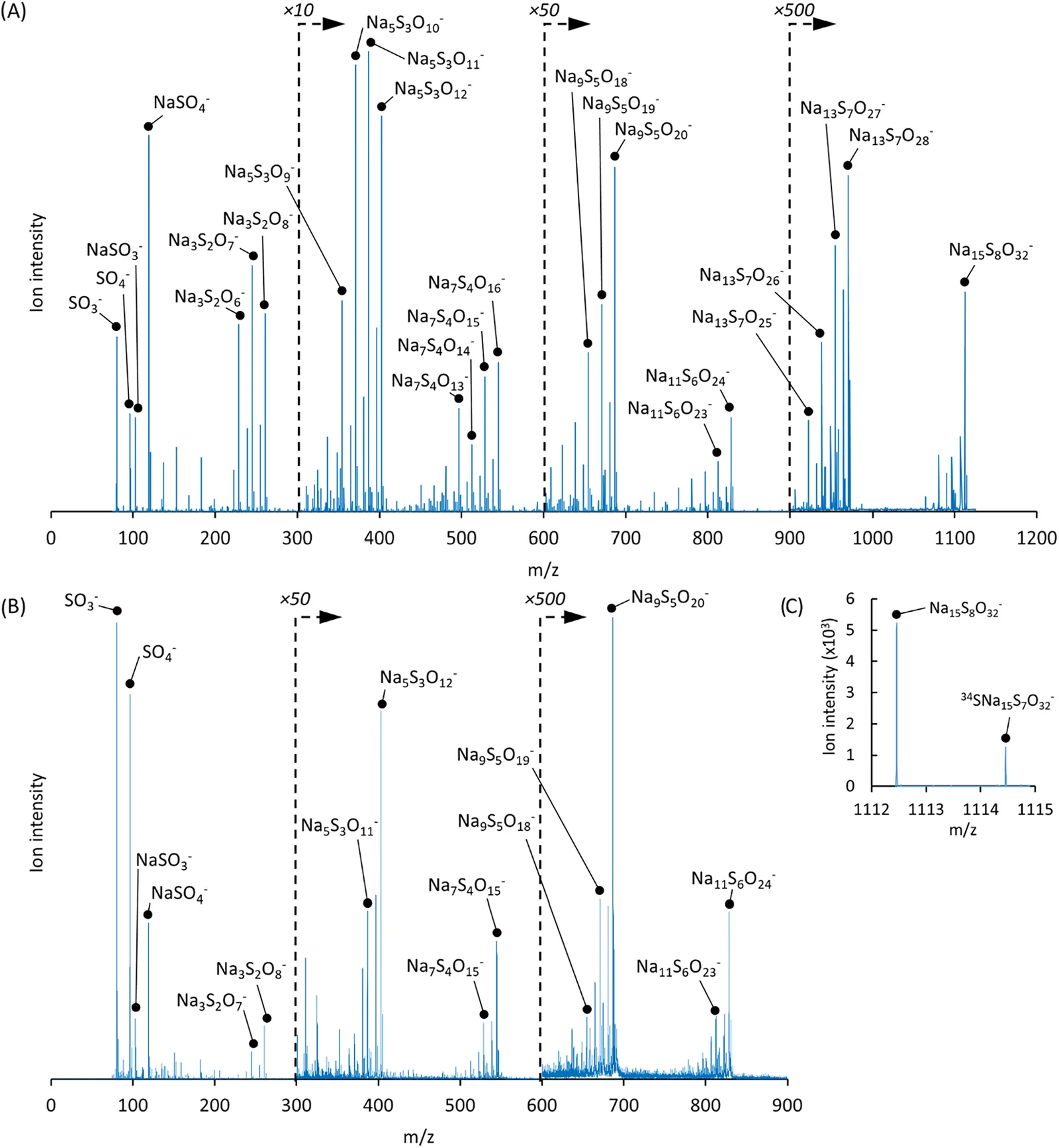
Representative SIMS spectra of PPS samples from manufacturer B taken using either an (A) Orbitrap or a (B) ToF mass analyzer. High-intensity ions assigned to sulfate clusters are labeled. A multiplication factor has been applied to the ion intensity to improve visualization, as indicated. (C) Zoomed mass range of OrbiSIMS analysis of PPS showing the Na_15_S_8_O_32_^–^ ion (ion count = 22772) and corresponding ^34^S isotope (ion count = 5549).

Clusters of high-intensity peaks with a spacing of a single oxygen were prominent for both the OrbiSIMS and ToF-SIMS spectra for all samples. The mass interval between clusters corresponds to the mass of sulfur. The presence of sulfur was confirmed through the presence of a corresponding ^34^S-containing ion in all cases (an example is shown in Figure 4C). These ions were assigned as sodium-sulfate clusters (deviation of less than 2 ppm). These ions are likely derived from NaSO_4_ salts that form upon drying of the sample but may also incorporate sulfate pendant groups on PPS. A strong association of sulfated GAGs with cations has been previously demonstrated.^34–36^ We note that the spectra of the PPS samples contain approximately 50 high-intensity (likely due to their relatively high ion yield) sodium-sulfate cluster ions, with >500 ions remaining that are likely derived from the PPS sugar structure including carbon-rich ions such as 71.02 (C_3_H_3_O_2_^–^), 115.00 (C_4_H_3_O_4_^–^), and 210.02 (NaC_7_H_7_O_6_^–^).

A comparison of the OrbiSIMS and ToF-SIMS spectra for the same PPS sample showed direct overlap in most cases. Consistent spectral data from tissue sections using matrix-assisted laser desorption ionization mass spectrometry imaging has been previously observed when switching between a ToF and a Fourier-transform ion cyclotron resonance mass analyzer.^37^ The improved mass resolution of the OrbiSIMS resolved additional ions that were indistinguishable with the mass resolution of the ToF mass analyzer. Over the mass range of *m*/*z* 80–250, 226 ions were identified in the ToF-SIMS spectrum compared with 488 in the OrbiSIMS spectrum. There were four typical cases observed for overlapping ToF-SIMS/OrbiSIMS peaks: (1) An apparent single ToF peak overlapped with a single Orbitrap peak (Figure 5A); (2) a ToF peak with a shoulder overlapped with two Orbitrap peaks whose mass and intensity matched what would have been predicted from the ToF peak (Figure 5B); (3) a ToF peak overlapped with multiple Orbitrap peaks (Figure 5C); and (4) a ToF peak with no corresponding Orbitrap peak (Figure 5D). The last case was observed 8 times. Cases where apparent shoulder peaks were present in the ToF-SIMS spectrum but had no corresponding OrbiSIMS peak were not included in this analysis. The loss of a small number of ions detected by ToF-SIMS when performing an OrbiSIMS analysis likely occurred during the relatively long ion transfer to the mass analyzer,^28^ likely through the decay of metastable ions.^38^

**Figure 5:**
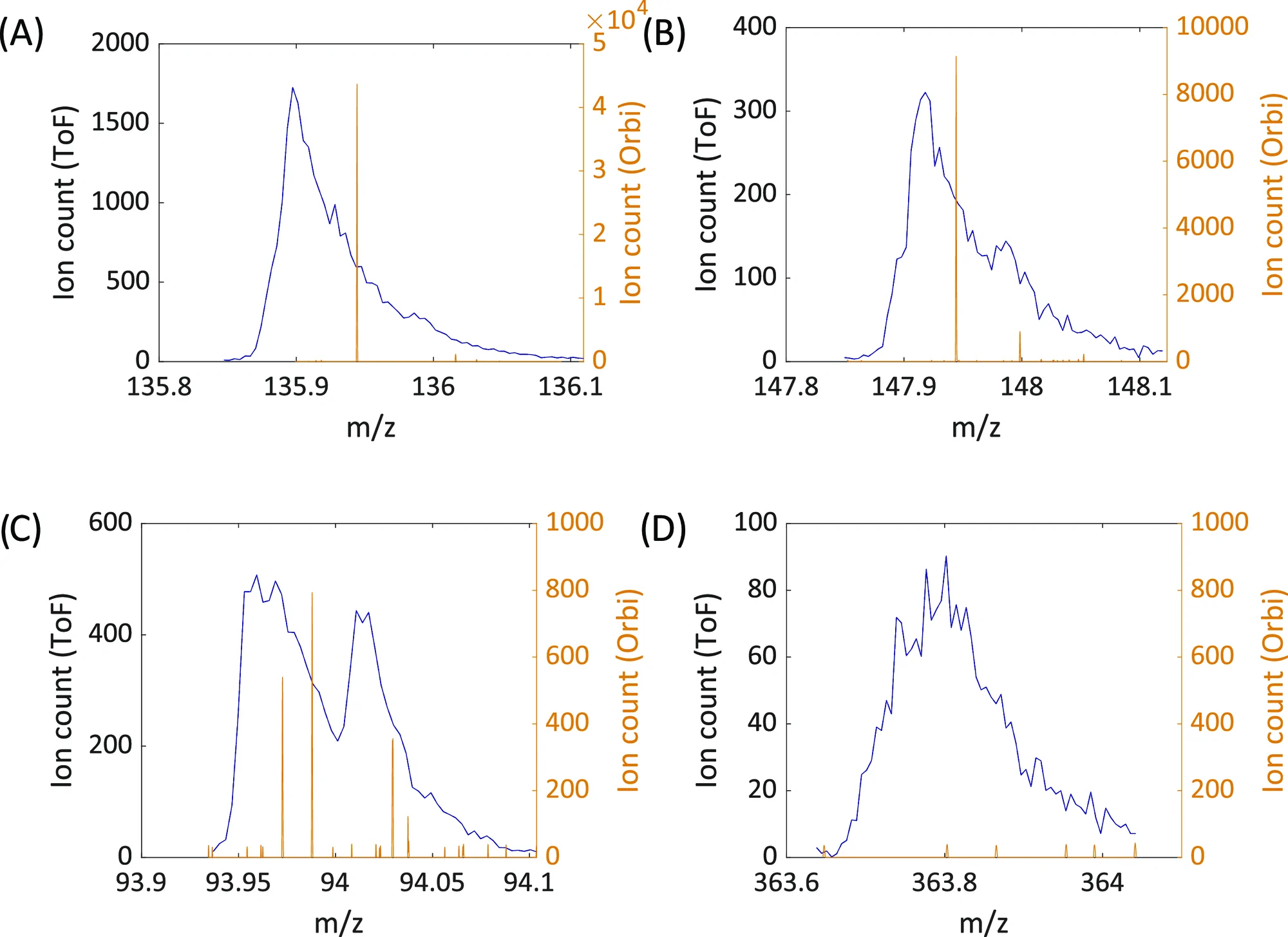
Comparison of the ToF-SIMS (Blue) and OrbiSIMS (Orange) spectra of PPS from manufacturer B, showing (A–C) examples of the mass-resolving power of the two mass analyzers for different peaks, or (D) an example of a peak present in the ToF-SIMS spectrum but not the OrbiSIMS spectrum.

### 3.3 Multivariate Analysis of SIMS Data

Multivariate analysis of the ToF-SIMS spectra of the PPS microarray was conducted to assess the discriminative power of the ToF-SIMS analysis for batch-to-batch and manufacturer-to-manufacturer variations. ToF-SIMS data were used due to the ease of commercial translation (ToF-SIMS can acquire data more quickly and there is a comparative abundance of ToF-SIMS instruments compared with OrbiSIMS instruments). The peak area associated with a peak list previously generated from a range of GAG samples^26^ was quantified. This list was used to remove any peaks likely derived from contaminants and to enable easy comparison with other GAG analyses;^26,27^ however, this list could be expanded to include chemical fragments that result from the unique chemical structures of PPS such as the branching. This was not required for the present study. PCA was performed, splitting the technical replicates 15:15 into training and validation datasets. A sparse dataset was created using RFA, maximizing the separation between samples, and then RFE, maximizing the confidence ellipses not overlapping, to remove uninformative descriptors, improve the discriminative power of the PCA model, and minimize overfitting.^39^ A total of 26 ions were selected as the minimum number of ions within 5% of the maximum mean area fraction not overlapping achieved (Figure 6A).

**Figure 6:**
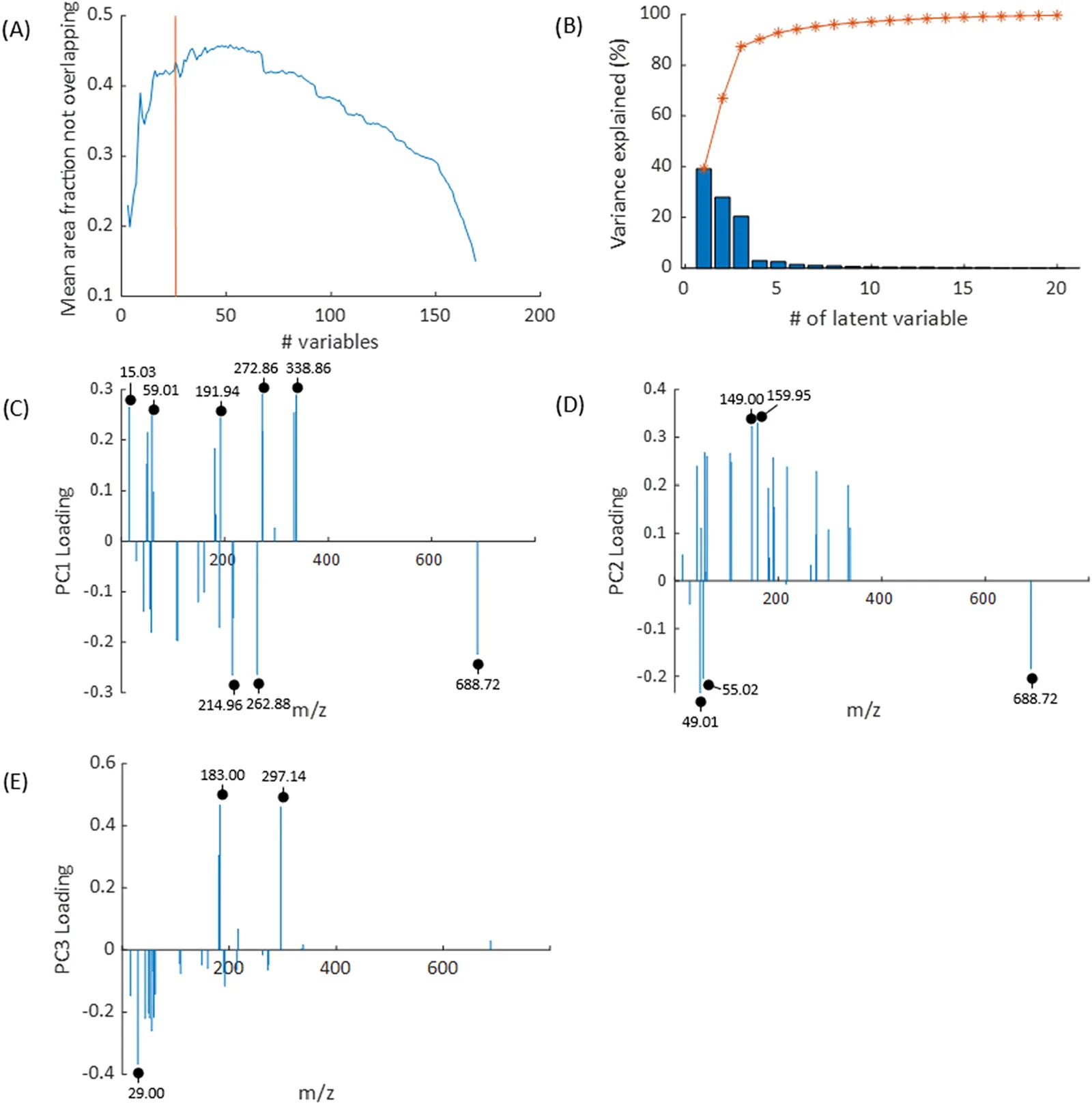
PCA model describing differences between PPS samples. (A) Mean area fraction of ellipses not overlapping used as cost function for RFE to select sparse dataset. Total number of ions selected (26) indicated by vertical orange line as minimum number of ions within 5% of maximum value measured. (B) Variance explained (cumulative line plot and for each latent variable as a bar chart) for varied number of latent variables. (C–E) PCA loadings with *m*/*z* of key ions indicated for (C) PC1, (D) PC2, and (E) PC3.

The resultant PCA model for the sparse dataset is shown in Figure 6. The first 3 principal components captured nearly 90% of the total variance, and each PC individually captured more than 20% of the variance (Figure 6B). Higher PCs captured minimal variance, thus, only the first 3 PCs were considered further. The loadings for the 26 ions used in the model for PCs 1–3 are shown in Figure 6C–E. It should be noted that these ions have not been selected due to their pharmaceutical relevance but rather in order to establish a methodology that can provide a rapid and reliable assessment of the similarities and differences between PPS batches.

The scores of validation datasets fell within the 95% confidence ellipses generated from the training datasets for all samples for PCs 1–3 (Figure 7A–C), suggesting that the model had not been overfitted. Considering the scores for PC1 and PC2, clear separation between samples from different manufacturers was observed (Figure 7A). For manufacturers A and C, different batches were undistinguishable (Figure 8A,C); however, for manufacturer B, each of the different batches could be separated to 95% confidence (Figure 8B). PC1 predominantly captured variance between manufacturers A and B and batch-to-batch difference for manufacturer B, while PC2 captured variance associated with differences between manufacturer C with the other manufacturers (Figure 7A).

**Figure 7:**
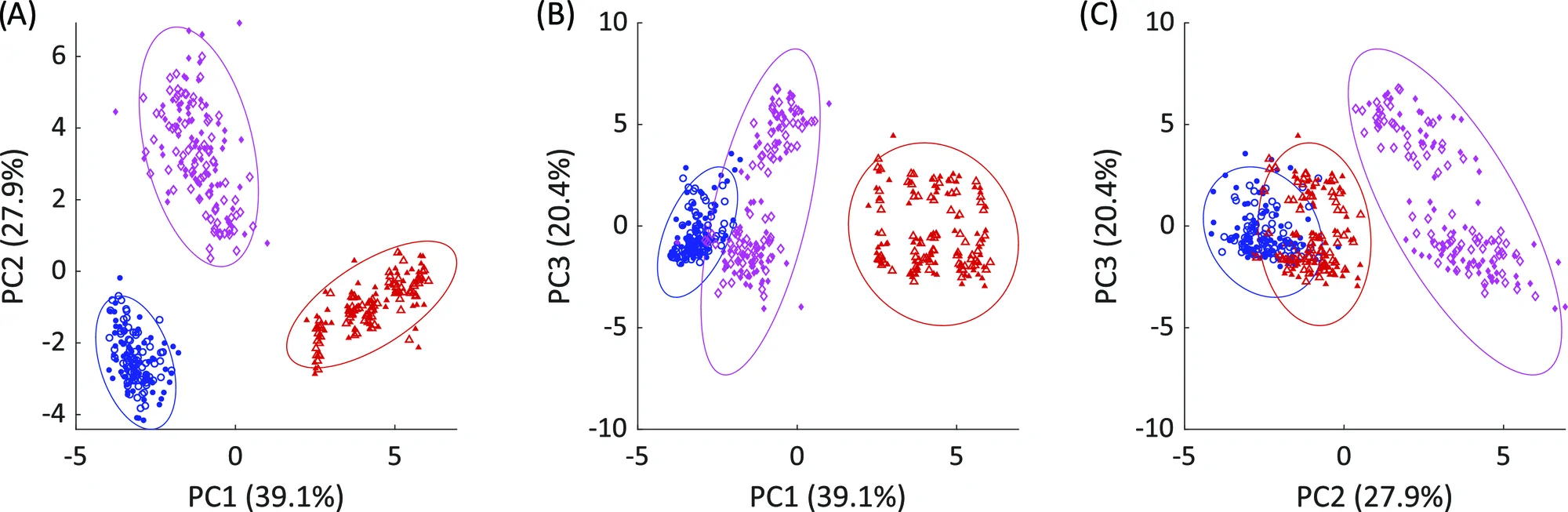
PCA scores plots for (A) PC1 and PC2, (B) PC1 and PC3, and (C) PC2 and PC3 for PPS samples from different manufacturers A (blue circle symbol), B (red triangle symbol), and C (pink diamond symbol). For each sample, 95% confidence ellipses are shown associated with 75 technical replicates. 150 technical replicates were assessed from each sample and were split 75:75 into training (closed symbol) and validation (open symbol) datasets.

**Figure 8:**
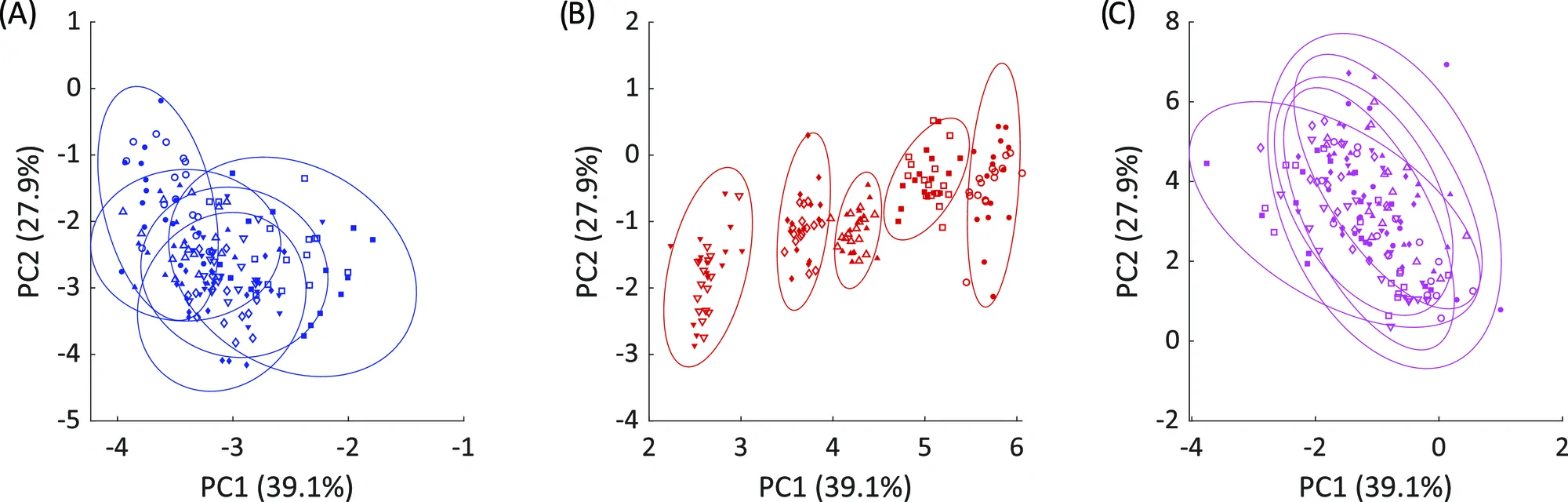
PCA scores plots for PC1 and PC2 for different batches of PPS from manufacturers (A) A, (B) B, and (C) C. For each sample, 95% confidence ellipses are shown associated with 15 technical replicates. 30 technical replicates were assessed from each batch sample and were split 15:15 into training (closed symbol) and validation (open symbol) datasets. Different batch samples are indicated by different symbols (●, ▲, ◆, ▼, ■).

Samples for manufacturers B and C were observed to split into 2 clusters for PC3 (Figure 7B,C). Multiple technical repeats of the same PPS batch sample were present in both clusters, suggesting that the variance captured by PC3 was associated with differences in technical replicates and could be attributed to different charging across the sample, or the presence of surface contaminants/inhomogeneities on the substrate surface.^27^

Each ion was assigned a loading for each PC, the magnitude of which indicated how an ion contributed to that PC. The PC loadings were thus investigated to explain the chemical functionalities associated with each PC. To aid this, the OrbiSIMS analysis of reference samples was used to provide high-confidence, putative assignments to the different ions used in the model. In some cases, multiple ions contributed to a single ToF-SIMS peak. Ions and their putative assignments are shown in Table 1, where all assignments are below 4 ppm deviation. The list of ions includes mostly small fragment ions such as CH_3_^–^, CHO^–^, and C_2_H_3_SO_3_^–^ with the number of carbons less than 5. The nonspecific nature of the ions limits the ability to identify the PPS structures associated with a particular PC. The inclusion of sulfur in many of these ions suggests an association with the sulfation pattern of PPS. This includes multiple ions with small carbon fragments connected to small sodium-sulfate clusters, suggesting an association of sulfate groups attached to PPS with the formation of sodium-sulfate cluster ions observed during SIMS analysis. The ion at *m*/*z* = 183.00, which can be assigned to C_8_H_7_SO_3_^–^, is likely associated with a singularly sulfated sugar unit. In some cases, high sodium adducts were observed (e.g., Na_4_C_4_H_4_O_3_^‑^), reflecting the high anionic charge of PPS. Thus, the ions present in the PCA model provide a general assessment of the structural characteristics of PPS, predominately associated with the degree of sulfation, with further structural interpretation limited by the complex molecular fragmentation associated with SIMS.

**Table 1:**
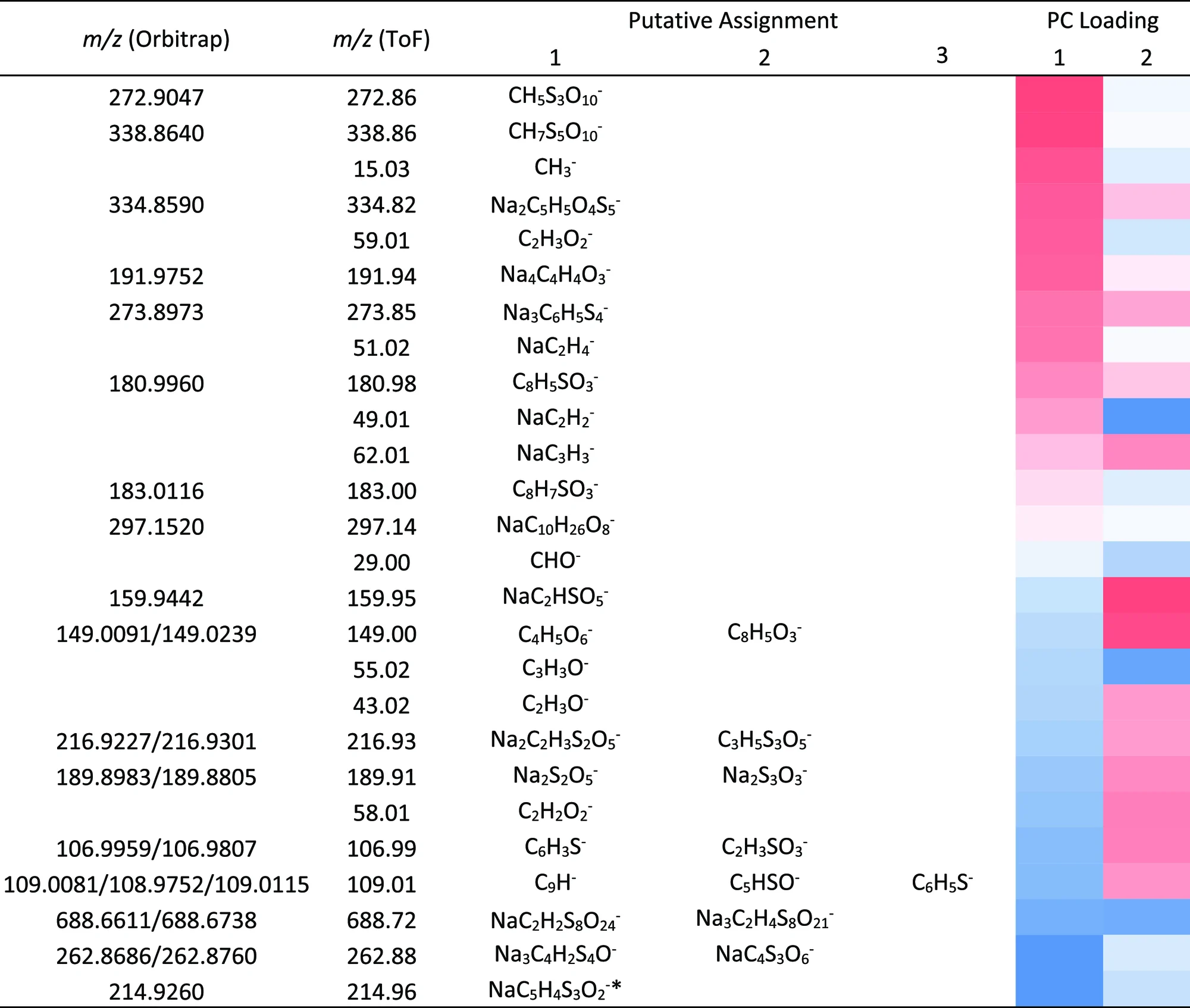
List of Selected Ions for the PCA Model Showing the Orbitrap and ToF *m*/*z* Values with Putative Assignments Based upon the Corresponding OrbiSIMS Spectra (<2 ppm Deviation) and Loadings for PCs 1 and 2^a^

a Multiple OrbiSIMS *m*/*z* values are included where multiple OrbiSIMS ions overlapped with a single ToF-SIMS peak (different *m*/*z* values ordered based upon ion intensity from the largest to the smallest). Loading has been colored based upon its value where red = positive and blue = negative. The ion list has been ordered based upon PC1 loading. *Assignment deviation greater than 4 ppm (7.7 ppm). Poor assignment may be due to multi-charged ion.^27,40^

### 3.4 Analysis of Spiked PPS Samples

To provide further structural insight into key ions identified via PCA, three PPS samples, one from each manufacturer, were spiked with NaSO_4_ at concentrations of 2.5 or 10 mM and reanalyzed by ToF-SIMS.

The ion Na_2_S_2_O_5_^–^/Na_2_S_3_O_3_^–^, associated with a negative loading for PC1, was likely derived from a sulfate cluster. This may include inter- and intramolecular interactions between sulfate groups, which would also contribute to the presence of a high number of sodium adducts (Table 1), or may be associated with free sulfate within the sample. These ions were investigated after being spiked with NaSO_4_. Increasing amounts of free sulfate led to a decrease in the intensity of the ion Na_2_S_2_O_5_^–^/Na_2_S_3_O_3_^–^ (Figure 9). The reduction may be due to (a) a disruption of the inter- and intramolecular interactions between sulfate groups attached to PPS chains by free sulfate or (b) a change in the ion yield from sulfate clusters as a result of the altered matrix, and likely indicates that the presence of sulfate clusters is not predominantly caused by free sulfate. The relationship between the production of sulfate cluster ions and free sulfate/sulfate residues is unclear.

**Figure 9:**
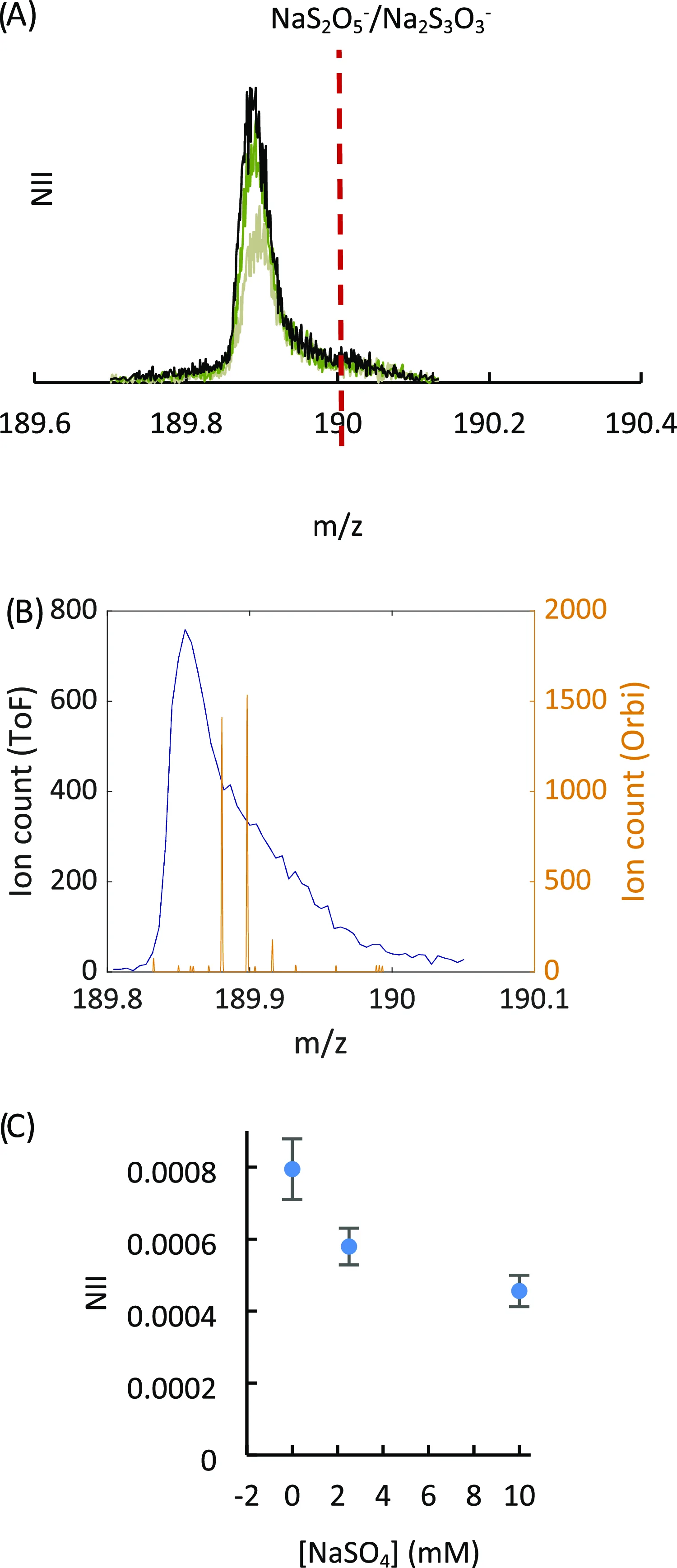
ToF-SIMS normalized ion intensity in PPS samples spiked with NaSO_4_. (A) Spectral data at *m*/*z* values for NaS_2_O_5_^–^/Na_2_S_3_O_3_^–^ for PPS B3 spiked with 0 (black square), 0.0025 M NaSO_4_ (green square) or 0.01 M NaSO_4_ (gray square). Spectral data given is the average of 9 technical replicates. The theoretical mass for the assigned ion is shown as the red dotted line. (B) Overlap of the ToF-SIMS (Blue Square) and OrbiSIMS (Yellow Square) spectra of PPS from manufacturer B at *m*/*z* = 189.9. (C) Normalized ion intensity for ions NaS_2_O_5_^–^ for PPS samples spiked with NaSO_4_. Error bars equal ± 1 SD, *N* = 3.

## 4 Conclusions

Discriminating between batch-to-batch as well as manufacturer-to-manufacturer differences in PPS samples is challenging due to the complex nature of these molecules but is critical for the production of PPS as an API. Use of high-throughput liquid handling and ToF-SIMS analysis enabled the rapid chemical analysis (450 samples in 6 h) of small amounts (≈150 ng) of PPS, with the ability to distinguish between PPS produced by different manufacturers and, in one case, batch-to-batch variation of the same manufacturer. A notable feature of the SIMS spectra of PPS samples was a high yield of sulfate cluster ions. Addition of free sulfate to PPS samples did not increase the yield of sulfate cluster ions, suggesting these ions are associated with PPS-bound sulfate, likely as a result of inter- and intramolecular interactions between PPS chains.

Samples also underwent NMR analysis to confirm their identity, which required ≈50 mg of sample and an analysis time of 5 h per sample due to the heterogeneous nature of PPS. This demonstrated the complementary nature of the two approaches. NMR provided detailed structural characterization but required relatively large samples amounts and analysis times. ToF-SIMS analysis provided rapid and high sensitivity discrimination between small amounts of samples; however, it was limited in the structural insights it could provide. These analysis attributes are of particular utility for quality control purposes and could aid in developing the safe use of PPS for additional clinical applications.

## Data Availability

Data for this article is available at the University of Nottingham data repository DOI = 10.17639/nott.7285.
